# Field assessment of dried *Plasmodium falciparum* samples for malaria rapid diagnostic test quality control and proficiency testing in Ethiopia

**DOI:** 10.1186/s12936-014-0524-z

**Published:** 2015-01-21

**Authors:** Afework Tamiru, Lucy Boulanger, Michelle A Chang, Joseph L Malone, Michael Aidoo

**Affiliations:** Nekemte Regional Public Health Laboratory, Oromia Regional Health Bureau, Addis Ababa, Oromia Ethiopia; Ethiopia Field Epidemiology Training Programme, Addis Ababa, Ethiopia; President’s Malaria Initiative, Centers for Disease Control and Prevention (CDC), 1600 Clifton Road, Atlanta, GA 30333 USA

**Keywords:** Malaria, Quality control, Rapid Test, Proficiency testing, Ethiopia

## Abstract

**Background:**

Rapid diagnostic tests (RDTs) are now widely used for laboratory confirmation of suspected malaria cases to comply with the World Health Organization recommendation for universal testing before treatment. However, many malaria programmes lack quality control (QC) processes to assess RDT use under field conditions. Prior research showed the feasibility of using the dried tube specimen (DTS) method for preserving *Plasmodium falciparum* parasites for use as QC samples for RDTs. This study focused on the use of DTS for RDT QC and proficiency testing under field conditions.

**Methods:**

DTS were prepared using cultured *P. falciparum* at densities of 500 and 1,000 parasites/μL; 50 μL aliquots of these along with parasite negative human blood controls (0 parasites/μL) were air-dried in specimen tubes and reactivity verified after rehydration. The DTS were used in a field study in the Oromia Region of Ethiopia. Replicate DTS samples containing 0, 500 and 1,000 parasites/μL were stored at 4°C at a reference laboratory and at ambient temperatures at two nearby health facilities. At weeks 0, 4, 8, 12, 16, 20, and 24, the DTS were rehydrated and tested on RDTs stored under manufacturer-recommended temperatures at the RL and on RDTs stored under site-specific conditions at the two health facilities. Reactivity of DTS stored at 4°C at the reference laboratory on RDTs stored at the reference laboratory was considered the gold standard for assessing DTS stability. A proficiency-testing panel consisting of one negative and three positive samples, monitored with a checklist was administered at weeks 12 and 24.

**Results:**

At all the seven time points, DTS stored at both the reference laboratory and health facility were reactive on RDTs stored under the recommended temperature and under field conditions, and the DTS without malaria parasites were negative. At the reference laboratory and one health facility, a 500 parasites/μL DTS from the proficiency panel was falsely reported as negative at week 24 due to errors in interpreting faint test lines.

**Conclusions:**

The DTS method can be used under field conditions to supplement other RDT QC methods and health worker proficiency in Ethiopia and possibly other malaria-endemic countries.

**Electronic supplementary material:**

The online version of this article (doi:10.1186/s12936-014-0524-z) contains supplementary material, which is available to authorized users.

## Background

Malaria continues to be a major cause of morbidity and mortality worldwide; over 40% of the world’s population, more than 3.3 billion people, live in countries with ongoing transmission and remain at risk [1]. In the 2013 World Malaria Report, it was estimated that in 2012 there were 207 million cases of malaria and 627,000 deaths, of which 90% occurred in sub-Saharan Africa [2].

Accurate diagnosis and treatment of malaria is critical in preventing severe manifestations and mortality of the disease and for disease control. The World Health Organization (WHO) revised malaria treatment guidelines in 2010, emphasizing the accurate parasitological confirmation of any suspected malaria fever case before prompt anti-malarial drug administration [3]. The revised WHO recommendation was aimed at decreasing the number of inappropriate prescriptions of anti-malarial drugs in order to preserve anti-malarial drug efficacy and to promote the appropriate treatment of patients presenting with febrile diseases of other aetiology. Critical to this revised policy is the availability of quality-assured confirmatory malaria diagnostic tests to guide malaria treatments.

Based on its performance characteristics, ease of use by non-laboratory staff working in health posts without laboratory facilities and in communities, rapid diagnostic tests (RDTs) have become a useful alternative to microscopic examination of thick blood films [4-9] for detection of infection, although thin film microscopy is still required for species identification when appropriate. Malaria RDTs can perform to a level similar to that provided by high-quality thick blood smear microscopy, can be introduced into peripheral health facilities and also be effectively used by community health workers [10-13] in remote rural locations that lack laboratory capacity for microscopy. The introduction of malaria RDT can also be useful in encouraging the rational use of expensive and sometimes limited availability of anti-malarial drugs [14-16].

Despite their utility, concerns about inter- and intra-lot variation of malaria RDTs and their susceptibility to high temperatures and humidity [17] require that RDT introduction is accompanied by a robust quality assurance/quality control (QA/QC) system to monitor test performance. Currently, the WHO, in collaboration with the US Centers for Disease Control and Prevention and the Foundation for Innovative New Diagnostics, conducts annual malaria RDT evaluations that monitor manufacturing quality of many of the widely used RDT products [17]. In addition to the product evaluation, WHO/FIND provides a free, pre-procurement, lot-testing service for countries that purchase RDTs; this service is routinely utilized by donors, including the Global Fund to Fight HIV/AIDS, Tuberculosis and Malaria and the US President’s Malaria Initiative. The product evaluation and pre-procurement lot-testing provide a quality system for RDTs prior to the tests being deployed in the field.

The RDTs distributed in many malaria-endemic countries may be exposed to higher temperatures than recommended by manufacturers [18]; in Ethiopia, the maintenance of a logistical supply chain with relatively cool ambient conditions may be especially difficult since items might be stored for days or weeks within shipping containers, and many items are imported through the port of Djibouti where very high temperatures are prevalent. A major challenge to widespread RDT use in the field is the ability to monitor their continued performance after exposure to the high temperature conditions typical of many malaria-endemic countries. In Ethiopia, a WHO/FIND certified malaria RDT testing laboratory is available at the Ethiopian Public Health Institute in Addis Ababa, but malaria infections are infrequently reported in Addis Ababa, and standardized reagents for quality control of RDTs have been unavailable previously.

The three current methods for monitoring RDT performance in the field include making blood smear microscopy slides in parallel with a subset of RDTs [12,19], directly observing malaria RDTs being performed and asking health workers to interpret pictorial representations of test results. These last two methods only assure the correct performance and interpretation of the test by the health worker and not the performance characteristics (sensitivity, specificity, positive, and negative predictive values) and accuracy of the RDT test over time. Preparing and examining blood smear slides depends on the availability of capacity for good quality microscopy and places additional burden on QA/QC for malaria microscopy. However, the major drawback to using smear microscopy to QC RDTs is that microscopy detects intact parasites while RDTs detect circulating parasite antigens. The parasite antigens detected by most malaria RDTs, histidine rich protein 2 (HRP2) and to a lesser extent plasmodium lactate dehydrogenase (pLDH) are known to persist for several days after parasite clearance and therefore limit the usefulness of microscopy for RDT QC [20]. Currently, no easily implementable method for RDT QC exists at central reference laboratories or in rural health facilities and such methods are needed to support quality universal diagnosis of malaria in remote locations.

RDTs are primarily designed for use on fresh capillary whole blood. Therefore, the QC sample needs to be a standardized, fresh and well-characterized clinical specimen, but significant challenges exist associated with sample variability and preservation [21]. Efforts at developing suitable recombinant antigens for QC testing [22] have so far been unrealized.

A method for RDT QC using dried *Plasmodium falciparum* infected blood samples in tubes has been reported [23]. These dried tube specimens (DTS) had stable RDT reactivity for over 52 weeks in the laboratory when stored under refrigeration (4°C) conditions, but needed to be further tested under field conditions. Factors that may influence the practicability of assembling DTS into a proficiency-testing panel to determine health workers’ ability to use and interpret RDT results were also assessed.

## Methods

The DTS were prepared as described [23]. Briefly, culture-grown *P. falciparum* parasites (strain 3D7) were diluted using haematocrit-matched parasite negative blood (0 parasites/μL) washed in RPMI1640 culture medium (Life Technologies, Grand Island, NY, USA) to concentrations of 500 or 1,000 parasites/μL. After testing for reactivity on replicate RDTs, 50 μL aliquots of each dilution (0, 500 and 1,000 parasites/μL) were transferred into open skirted base, conical-bottomed Sarstedt® Type I micro tubes (Sarstedt Inc, Newton, NC, USA) and air dried in a biosafety cabinet. To test for retention of reactivity after drying, one tube each of 0, 500 and 1,000 parasites/μL DTS was rehydrated in PBS-Tween solution as previously described [23] and tested on the same RDT brand as that used prior to drying. After verifying baseline RDT reactivity, the DTS were capped and stored in a 4°C refrigerator until transported to Addis Ababa, Ethiopia in November 2012.

### Study setting (site selection)

The regional reference laboratory, Adama Regional Reference Laboratory (ARRL), about 110 km southeast of Addis Ababa within the Oromia Regional State, was selected as the reference laboratory due to the availability of controlled temperature environments to store DTS at the recommended 4°C. The criteria for choosing the two peripheral health centres were the routine use of RDTs and the feasibility of RDT storage at ambient temperature. To simplify study logistics, the two health centres were selected in order for both to be visited on the same day and within eight hours of DTS reconstitution at the reference laboratory. Accordingly, Wanji Health Centre, located 10 km and Walanchiti Health Centre, located 27 km from ARRL and within the Great Rift Valley in the central part of Ethiopia were selected as the health centres for this study.

### RDT selection

The malaria RDT brand chosen for the study was the CareStart malaria pLDH/HRP2 combo test. These RDTs, with an expiry date of February 2015, were obtained from a recent shipment to the UNICEF central warehouse in Addis Ababa procured through the PMI and meant for distribution throughout Ethiopia. The CareStart malaria pLDH/HRP2 combo test contains a membrane strip pre-coated with two monoclonal antibodies, designed for the diagnosis of *P. falciparum* and/or other *Plasmodium* species, including *Plasmodium vivax*.

### DTS accession and field monitoring

DTS were prepared at CDC, Atlanta in advance, with aliquots of 0 parasites (negative), 500 and 1,000 parasites/μL and shipped on ice packs to Ethiopia. Because DTS were initially tested at baseline in Atlanta with different RDT brands from those used in Ethiopia, RDT reactivity was confirmed on the CareStart malaria pLDH/HRP2 combo RDT brand immediately upon arrival in Ethiopia. Following confirmation of reactivity, DTS were stored at 4°C at ARRL and under site-specific conditions at the two health centres. The CareStart Combo RDTs from the same lot as mentioned above were also distributed to these three study sites. According to the product insert supplied by the manufacturer, the CareStart Combo RDT has sensitivity and specificity for *P. falciparum* of 98 and 97.5%, respectively. However, the parasite density of the samples used in defining these test characteristics and the lower limit of detection of the test were not provided.

At time week zero and at four-week intervals over a period of six months, RDTs stored at ARRL under manufacturer-recommended storage conditions and the same lot/batch of RDTs stored at room temperature in the two health centres were tested for performance using DTS stored at 4°C at the ARRL and at room temperature in the peripheral health centres. DTS were rehydrated with the same volume of PBS/Tween 20 solution as initial blood volume and tested on RDTs within eight hours of rehydration.

### DTS field monitoring

#### Adama regional reference laboratory (ARRL) testing

On the day of testing, one aliquot each of DTS at 0, 500 and 1,000 parasites/μL stored at 4°C at the ARRL was rehydrated over one hour with two drops of PBS/Tween using a plastic Pasteur pipette (i.e., approximately 50 μL) and tested on duplicate RDTs stored under manufacturer-recommended storage conditions (≤30°C) at the reference laboratory. Field testing at the health centres was done on the same day. The ARRL site served as the gold standard for DTS and RDT optimal storage conditions and RDT test performance in Ethiopia while the two health centres represented peripheral facility level ambient conditions for comparison.

#### Field-testing

On the same day as ARRL testing, the rehydrated DTS and RDTs from ARRL were transported at ~4°C and manufacturer-recommended storage conditions (i.e., optimal conditions), respectively, to the first health centre. At the health centre, rehydrated DTS from ARRL and rehydrated replicate health centre-stored (room temperature) DTS were each tested on both RDTs brought from ARRL and health centre-stored RDTs. For the purposes of this evaluation, DTS were tested within eight hours of rehydration.

#### Quality control

To avoid errors that could be attributed to the competence of health-worker performance and interpretation of RDT results, regional laboratory staff with experience in performing malaria RDTs were selected and provided with additional training. To rule out interpersonal variability, the same person was appointed for the whole study period. In each month the collected data were checked by the investigators for any transcriptional error resulting either while interpreting or summarizing the data from the facility reports.

#### Proficiency-testing panel

Four DTS tubes stored at 4°C at ARRL were assembled into blinded panels each consisting of one 0 parasites/μL (negative control), two 500 parasites/μL and one 1,000 parasites/μL DTS tubes for PT. During health centre visits at weeks 12 and 24, health workers at the facility were asked to perform RDT tests using this panel on RDTs stored under manufacturer-suggested conditions brought from ARRL. Using a checklist and without intervention, ARRL staff observed and scored performance of nine critical steps in RDT testing and result interpretation by health centre staff. Errors in test performance by health centre staff were corrected by ARRL staff after the proficiency-testing was completed.

#### Data collection procedures and management

The testing site name, week and test results of the three study sites were collected on a standard form every four weeks and then transferred to a single form. Proficiency panel test results were recorded on a separate form. The results were subsequently transferred into an electronic database, Microsoft Excel.

### Data analysis

The results obtained from ARRL were considered the gold standard by which to assess the DTS and RDT stored under field conditions in the two health centres. The concordance of ARRL results with the result from health centre-DTS tested on ARRL RDT was used to show the stability of health centre-stored DTS. The concordance of results of ARRL-stored DTS on the health centre-stored RDTs with ARRL-stored DTS on ARRL-stored RDTs determined the stability of health centre-stored RDTs.

### Ethical clearance

The protocol for this study was reviewed and approved by the Human Subjects Coordinators for the Division of Parasitic Diseases and Malaria and the Center for Global Health, Centers for Disease Control and Prevention, Atlanta. A full Institutional Review Board assessment was not required because no human or animal subjects were involved in the study.

## Results

### DTS testing

#### ARRL testing

At ARRL where both DTS and RDT were stored at the recommended temperatures, the DTS (at 0, 500 and 1,000 parasites/μL) retained their reactivity at all the seven testing time points for 24 weeks (Table 1). The expected results were obtained for four replicates of each parasite concentration of DTS at every time point (a total of 84 tests).

**Table 1:** **ARRL-stored DTS reactivity for each time point on four replicates of RDT stored at Adama regional reference laboratory**

**ARRL DTS**	**ARRL RDT result**						
	**Wk0**	**Wk4**	**Wk8**	**Wk12**	**Wk16**	**Wk20**	**Wk24**
0 P/μL	− − − −	− − − −	− − − −	− − − −	− − − −	− − − −	− − − −
500 P/μL	+ + + +	+ + + +	+ + + +	+ + + +	+ + + +	+ + + +	+ + + +
1,000 P/μL	+ + + +	+ + + +	+ + + +	+ + + +	+ + + +	+ + + +	+ + + +

− = Negative test result, + = positive test result.

#### Health facility testing

At both health centres the peripheral health centre-stored DTS at *P. falciparum* parasite concentrations of 0, 500 and 1,000 parasites/μL produced the expected results at all the seven time points (a total of 21 tests/facility) when tested on RDT transferred from ARRL (Table 2). Similar to the ARRL results, when tested on health centre-stored RDTs, the reconstituted DTS, the same samples as the three concentrations used at ARRL, were negative for 0 parasites/μL and positive for 500 and 1,000 parasites/μL at all time points (Table 2).

**Table 2:** **ARRL and health centre-stored DTS reactivity on health centre and ARRL-stored RDTs**

**Test panels**		**Results**																				
**DTS Storage Location**	**RDT Storage Location**	**Wk0**			**Wk4**			**Wk8**			**Wk12**			**Wk16**			**Wk20**			**Wk24**		
		**Parasites/μL**			**Parasites/μL**			**Parasites/μL**			**Parasites/μL**			**Parasites/μL**			**Parasites/μL**			**Parasites/μL**		
		**0**	**500**	**1,000**	**0**	**500**	**1,000**	**0**	**500**	**1,000**	**0**	**500**	**1,000**	**0**	**500**	**1,000**	**0**	**500**	**1,000**	**0**	**500**	**1,000**
ARRL	Walanchiti HC	-	+	+	-	+	+	-	+	+	-	+	+	-	+	+	-	+	+	-	+	+
Walanchiti HC	ARRL	-	+	+	-	+	+	-	+	+	-	+	+	-	+	+	-	+	+	-	+	+
Walanchiti HC	Walanchiti HC	-	+	+	-	+	+	-	+	+	-	+	+	-	+	+	-	+	+	-	+	+
ARRL	Wanji HC	-	+	+	-	+	+	-	+	+	-	+	+	-	+	+	-	+	+	-	+	+
Wanji HC	ARRL	-	+	+	-	+	+	-	+	+	-	+	+	-	+	+	-	+	+	-	+	+
Wanji HC	Wanji HC	-	+	+	-	+	+	-	+	+	-	+	+	-	+	+	-	+	+	-	+	+

− = Negative test result, + = positive test result, HC = health centre.

Health centre-stored DTS were also tested on health centre-stored RDTs. This health centre-specific testing was meant to demonstrate the stability of DTS under field specific storage conditions and their utility as QC samples for RDTs stored under the same conditions. In this testing, DTS at 0, 500 and 1,000 parasites/μL stored at room temperature in Walanchiti Health Centre and Wanji Gefersa Health Centre retained their reactivity on RDTs stored under the same conditions at all the time points from week 0 to week 24. Overall 21 tests per facility using DTS samples from each concentration retained their baseline reactivity (Table 2).

#### Proficiency-panel testing

Proficiency-panel testing results indicated that of the three study sites, only at Wanji Gefersa Health Centre were all samples in the panel scored correctly at the two panel testing points, week 12 and week 24. The other two study sites, ARRL and Walanchiti Health Centre reported false results for panel samples at 500 parasites/μL on week 24. At ARRL three out of four faint bands were reported as negative and at Walanchiti Health Centre, two of four faint bands were reported as negative at week 24. In total, 16 tests, four each of the four panel samples, were tested at each site at week 12 and week 24. Therefore, 16/16 panel samples at Wanji Gefersa Health Centre, 13/16 at ARRL and 14/16 at Walanchiti Health Centre were reported correctly at week 24 (Table 3).

**Table 3:** **Proficiency testing results of the ARRL, Wanji health centre and Walanchiti health centre**

**Study site**	**PT sample**	**Code**	**PT result**		**Remark/comment**
			**Week 12**	**Week 24**	
ARRL	Four 1,000 p/μL	001	+ + + +	+ + + +	
	Four 500 p/μL	002	+ + + +	+ + + +	
	Four 0 p/μL	003	− − − −	− − − −	
	Four 500 p/μL	004	+ + + +	+− − −	Faint test line missed
Wanji Health Centre	Four 1,000 p/μL	001	+ + + +	+ + + +	
	Four 500 p/μL	002	+ + + +	+ + + +	
	Four 0 p/μL	003	− − − −	− − − −	
	Four 500 p/μL	004	+ + + +	+ + + +	
Walanchiti Health Centre	Four 1,000 p/μL	001	+ + + +	+ + + +	
	Four 500 p/μL	002	+ + + +	+ + − −	Faint test line missed
	Four 0 p/μL	003	− − − −	− − − −	
	Four 500 p/μL	004	+ + + +	+ + + +	

− = Negative test result, + = positive test result.

While performing the DTS proficiency-testing panel, study site staff were assessed for procedural mistakes using a nine-step checklist (Table 4) completed by observing the technicians. The assessment showed that at week 12 one technician added excess blood than the test instruction specified. At week 24, technicians from Walanchiti Health Centre (same technician from week 12) and ARRL interpreted a faint positive band incorrectly as a ‘no band’ (Table 4) and scored the test as negative.

**Table 4:** **Proficiency testing checklist performed during supervisory visits showing sample result from the study sites**

**Step**	**Assessing questions**	**Technician’s performance**					
		**Proficiency testing Week 12**			**Proficiency testing Week 24**		
		**ARRL**	**Wan. HC**	**Wal. HC**	**ARRL**	**Wan. HC**	**Wal. HC**
1	Correct volume of blood collected	Yes	***No***	Yes	Yes	Yes	Yes
2	Correct amount of blood applied	Yes	***No***	Yes	Yes	Yes	Yes
3	Blood applied to sample well	Yes	Yes	Yes	Yes	Yes	Yes
4	Correct amount of buffer dispensed	Yes	Yes	Yes	Yes	Yes	Yes
5	Buffer dispensed to buffer well	Yes	Yes	Yes	Yes	Yes	Yes
6	Timer set for incubation	Yes	Yes	Yes	Yes	Yes	Yes
7	Correct time set for incubation	Yes	Yes	Yes	Yes	Yes	Yes
8	Results read at time specified by test	Yes	Yes	Yes	Yes	Yes	Yes
9	Results interpreted correctly	Yes	Yes	Yes	***No***	Yes	***No***

Wan = Wanji Health Centre, Wal = Walanchiti Health Centre.

## Discussion

Malaria RDTs are increasingly being used in most malarious countries in sub-Saharan Africa and are now distributed to peripheral health facilities including rural health posts, and to health centres without laboratories and sometimes to volunteers working in communities with minimal training. In these peripheral settings, it may be difficult to transport and to maintain RDTs within recommended storage temperature conditions [4]. One practical way of limiting the exposure of RDTs to high temperatures is to limit prolonged storage under extreme conditions, and to maintain a ‘cool’ supply chain, but it is often impractical or extremely difficult to determine that RDTs were never exposed to extreme environmental conditions at some time after manufacture. Previously, quality control of RDTs comprised simultaneous preparation of smear slides for microscopy along with a proportion of rapid tests. However, the methodology has several practical limitations and has largely been abandoned since RDTs detect parasite antigens as opposed to actual parasites by microscopy. Currently, the only viable, easily implementable methodology is to directly observe the use of RDTs at the facility during supervision and through health worker interpretation of photographs of RDT results. None of the methods mentioned above adequately determine reduction in RDT performance resulting from exposure to excessive temperature and humidity typical of malaria-endemic areas or other adverse conditions. The lack of an appropriate quality control method for RDT, especially the absence of positive control samples, might also undermine confidence in the accuracy of malaria RDT results by health workers [24,25]. Therefore, a practical method for malaria RDT QC on an ongoing basis remains critical to the widespread use of malaria RDTs in remote rural areas.

The potential for using dried culture-derived *P. falciparum-*infected blood as QC samples to identify failing malaria RDTs has been reported previously [23]. Also, additional experiments indicated that DTS at 1,000 and 2,000 parasites/μL and stored at 4°C were stable for up to two years ([23] and Additional file 1).

In the experiments described here, DTS stored under refrigeration in a reference laboratory and those stored at ambient temperatures under field conditions were stable for the seven-month duration of the study, repeatedly providing expected results. Storage temperatures in the health centres were not measured to determine whether there was significant temperature differences between the health centre- and the reference laboratory-stored DTS. In addition, ambient temperatures, although much higher than 4°C during that period in Ethiopia, were likely within manufacturer-recommended storage temperatures (4°C-30°C) according to weather data from the Ethiopian Meteorology Agency. Despite the potential differences in storage temperatures between the health centres and reference laboratory, there is no reason to believe they had any measurable effect on RDT performance. Ensuring that RDTs perform as expected is a major reason to have a QC method. Therefore, one of the uses of *P. falciparum* DTS as a standardized substrate for RDT testing is to assure health workers of the continued reliability of the RDT test results at their facility. The availability of QC data regarding RDTs could result in trust of RDT results and therefore fewer instances of over-treatment. In addition to QC testing, DTS were assessed for utility as proficiency-testing samples for health workers. Proficiency-testing is an important component of QA and having well-characterized samples for QC and proficiency-testing further enhances confidence that RDT testing as practiced by health workers in rural areas is providing reliable laboratory results that are leading to good case management of malaria. Together with a checklist, DTS were used to assess the competency of health professionals in performing RDTs, and in interpreting and reporting results. Proficiency testing administration provided an opportunity to identify and correct errors in reading faint positive test lines. While the possibility of sample decay at week 24 is a possible reason for the reading error, the fact that other technicians were able to correctly score that test result for that sample suggests the technician rather than band intensity as the reason for the error. Having DTS with parasite densities closer to the detection limit of the RDT may provide an opportunity to teach health workers test interpretation, especially where most problems with RDT test interpretation exist. The use of DTS might improve overall testing quality in the process.

In this study it is shown that well-characterized DTS stored either at facility level ambient temperature conditions or in a reference laboratory under refrigeration can be used for RDT QC and proficiency-testing in a field-implementable manner in Ethiopia and that the DTS are stable under such conditions for a period of at least seven months. These data complement and confirm reported controlled laboratory-based experiments in which DTS stored in a refrigerator remained stable for > two years (Additional file 1). This study did not provide any RDT testing data (except for universally negative results) that would be relevant to the detection of *P. vivax* infections in Ethiopia, since the DTS were prepared using *P. falciparum* only.

DTS has several benefits over current methodologies for RDT QC. First, observation of how tests are performed on actual patients requires that a suspected malaria case is present during the observation. However, in a country such as Ethiopia with relatively low malaria prevalence, such a situation is not guaranteed unless one spends a significant amount of time at the facility. Even when a case is available, the testing by a technician can be observed but without the benefit of expected reactivity of the sample, the quality of the test cannot be determined. Second, by having DTS with known reactivity, it is possible to monitor whether site-specific storage and transportation conditions could have compromised test performance. In addition, DTS can be used to adjust the density of infection in samples and therefore the degree of difficulty in interpreting results within a proficiency-testing programme.

## Conclusions

The ability of health workers to properly perform malaria RDTs (proficiency testing) and to monitor the quality of RDT test results can be assessed with cultured *P. falciparum* DTS as an RDT testing substrate in rural areas of Ethiopia. The DTS methodology can enhance the scale-up of quality RDT testing. Evaluation of DTS developed with well-characterized patient blood could also be explored in order to make the method widely available.

## Additional file

Additional file 1:
**Long-term testing of DTS stored at 4°C showing reactivity up to 109 weeks of storage.** Relative intensities of the control “C” and the parasite specific “Pf” bands are indicated by “+” with the number of “+”s denoting increasing band intensity.

## Abbreviations

ARRL: Adama Regional Reference Laboratory
DTS: Dried Tube Specimen
HRP2: Histidine-rich protein 2
pLDH: Plasmodium lactate dehydrogenase
QA: Quality assurance
QC: Quality control
RDT: Rapid diagnostic test
Wal. HC: Walanchiti Health Centre
Wan. HC: Wanji Health Centre
