# Supplementary material for: Field assessment of dried Plasmodium falciparum samples for malaria rapid diagnostic test quality control and proficiency testing in Ethiopia
Source: Malar J. 2015 Jan 21;14:11. doi: 10.1186/s12936-014-0524-z (PMC4320833; doi:10.1186/s12936-014-0524-z)

**Additional File 1**

Long-Term testing of DTS stored at 4^o^C showing reactivity up to 109 weeks of storage. Relative intensities of the control “C” and the parasite specific “Pf” bands are indicated by “+” with the number of “+”s denoting increasing band intensity.


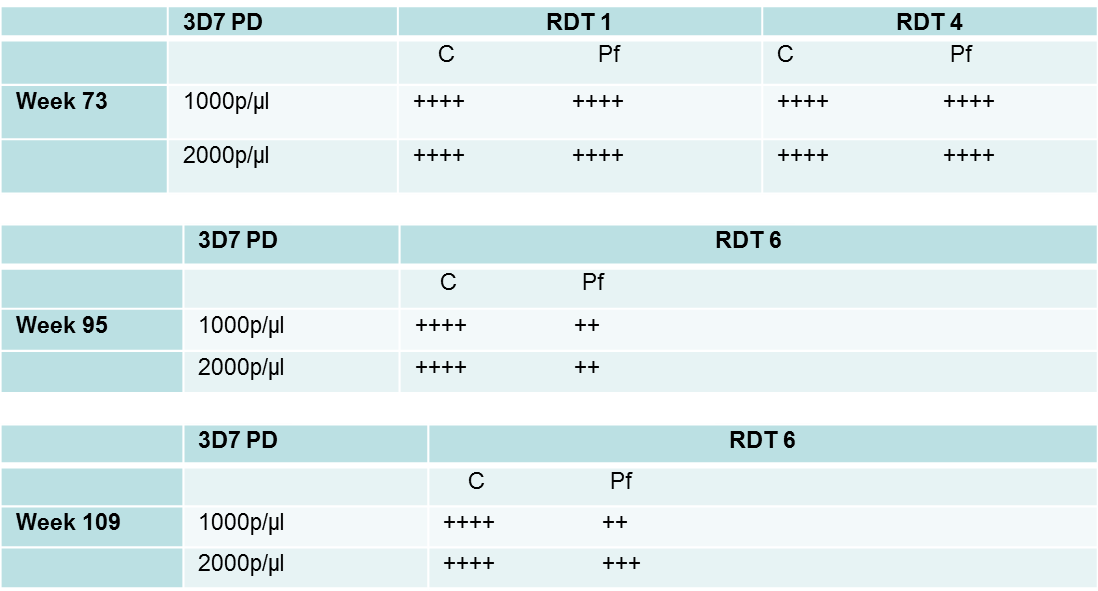

Supplement: Additional file 1: — Long-term testing of DTS stored at 4°C showing reactivity up to 109 weeks of storage. Relative intensities of the control “C” and the parasite specific “Pf” bands are indicated by “+” with the number of “+”s denoting increasing band intensity. [file 12936_2014_524_MOESM1_ESM.docx]
